# Case Report: Magnetic Resonance Imaging Features of Scrotal Angiomyofibroblastoma (AMF) With Pathologic Correlation

**DOI:** 10.3389/fsurg.2022.808488

**Published:** 2022-04-29

**Authors:** Jing Zeng, Lingtao Zhang, Changzheng Shi, Liangping Luo

**Affiliations:** ^1^Medical Imaging Center, The First Affiliated Hospital of Jinan University, Guangzhou, China; ^2^Engineering Research Center of Medical Imaging Artificial Intelligence for Precision Diagnosis and Treatment, Guangzhou, China

**Keywords:** angiomyofibroblastoma (AMF), magnetic resonance imaging, angiomyofibroblastoma-like tumor, aggressive angiomyxoma, case report

## Abstract

Angiomyofibroblastoma (AMF) is a rare benign myofibroblastic tumor that mainly occurs in the genital tract of middle-aged female patients. However, it can also arise in the scrotum, spermatic cord, and bladder. We described, herein, a case of a 42-year-old patient who was admitted to our hospital with a left scrotal mass. Imaging examinations showed that the mass had abundant vessels and displayed obvious progressive intensification on enhanced MRI. The following histopathological and immunohistochemical studies led to the diagnosis of AMF. Here, we describe the magnetic resonance imaging findings of a case of scrotal AMF. We hope that the information can help radiologists to identify AMF.

## Introduction

Angiomyofibroblastoma (AMF) is a rare tumor. At present, due to the paucity of AMF, the literature is mainly limited to case studies and there is no statistical data on its incidence. In addition, the genetic and ethnic differences of this tumor have not been calculated. Though AMF is a rare tumor that most commonly affects women (1), it also occurs in men and can arise in the inguen, scrotum, spermatic cord, and bladder (2). Only 23 cases of male AMF were reported in the English-language literature based on a review of PubMed data since 1992 (3). We also found that the MRI performances of AMF in men have rarely been reported. Here, we describe an additional case of scrotal AMF and its MRI findings. We hope that the imaging features of AMF reported can supplement the content in this aspect.

## Case Description

A 42-year-old man with a left swelling scrotum was admitted to our hospital. The mass had remained the same in size for 4 months and the patient could feel slight tenderness in his left scrotum. He reported no vomiting, fever, or abdominal pain. His personal histories were unremarkable, and there was no family history of genetic disease.

**Figure 1:**
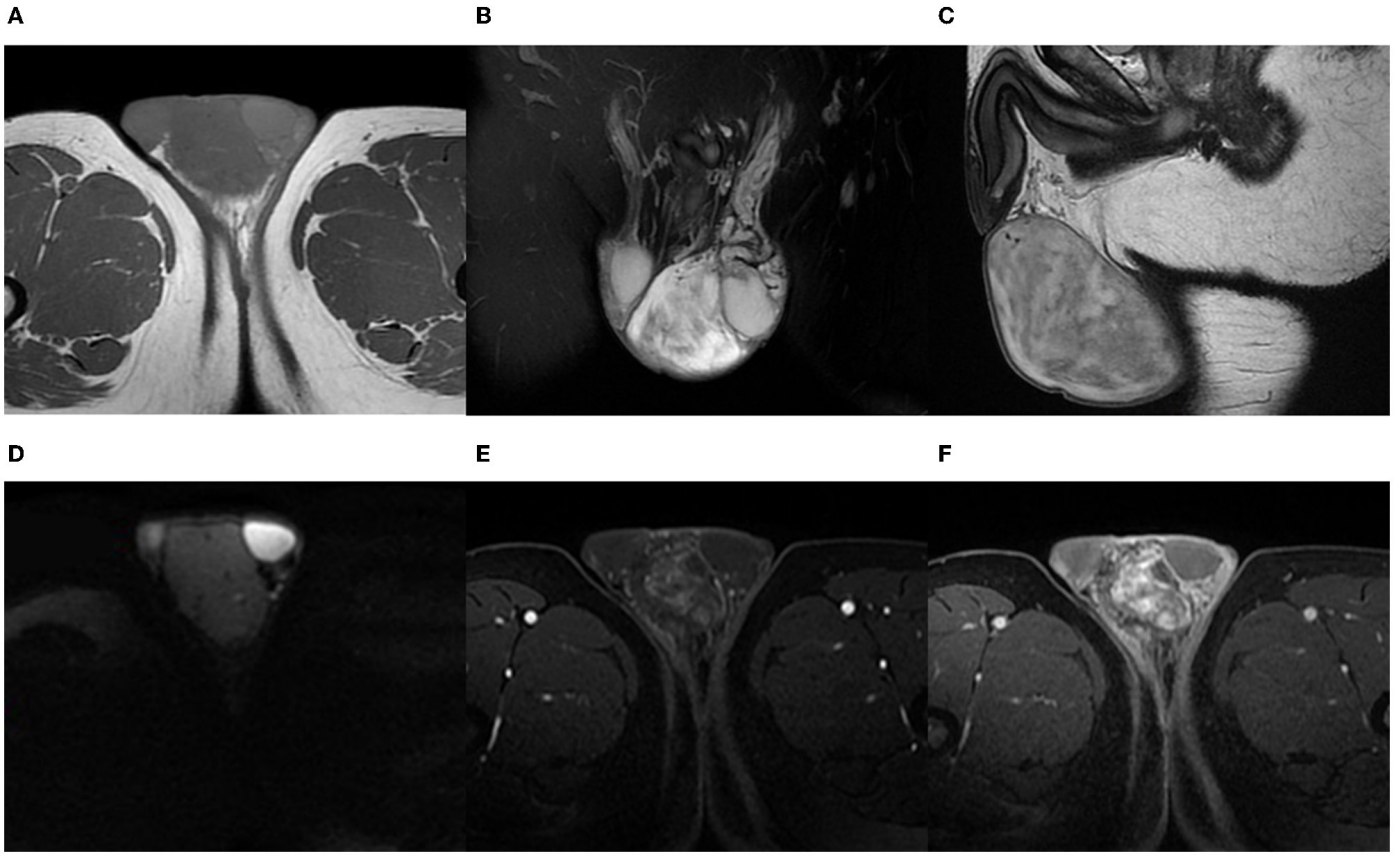
A 42-year-old man with AMF. On T1-weighted images, the tumor reveals low signal intensity but high signal intensity in some circuitous strips **(A)**. On coronal fat-saturated T2-weighted images and sagittal T2-weighted images, the tumor is well-marginated with high inhomogeneous signal intensity **(B,C)**. DWI shows a low-signal tumor **(D)**. The images of contrast-enhanced MRI show a heterogeneous and distinctly enhanced mass. As time goes on, the enhancement of the tumor is more obvious **(E,F)**.

On his physical examination, a 6 cm × 5 cm mass was palpated in his left scrotum with a smooth surface. However, the left testicle was unpalpable. The indicators in his laboratory inspections were within the normal range. Then to find out the internal structure of the tumor, he underwent an MRI examination. A tumor was confined to the left scrotal wall, adjacent to the testicle and epididymis. The mass displayed a well-defined border with the left testicle. It appeared hypointense with few vascular flow empty shadows and hypersignal areas on T1-weighted images (Figure 1A). On T2-weighted images on the MRI scan, the tumor was heterogeneous hyperintense and well-marginated (Figures 1B,C). The mass showed no restricted diffusion on diffusion weighted imaging (DWI) (Figure 1D). Contrast-enhanced MRI images showed a non-homogenous but markedly enhanced mass with progressive enhancement (Figures 1E,F). On the MRI examination, no abnormal signals were seen in the left testicle, indicating that the tissues around the tumor have not been involved. We can also see the left spermatic vein was tortuous and widened. According to the MRI results, it was thought of as a low-grade malignant tumor and derived from non-germinal cells. After that, an ultrasound examination was performed, and the mass was considered to be a germ cell-derived tumor, which was contrary to the diagnosis of radiologists.

**Figure 2:**
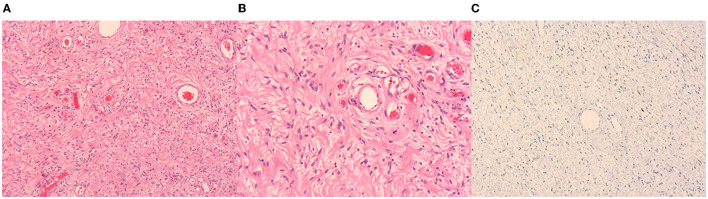
Hematoxylin-eosin (HE) staining and immunohistochemistry of the tumor. Thin-walled blood vessels surrounded by several spindle cells can be seen in the images **(A,B)**. Image A and Image B represent 50× and 100×, respectively. The desmin is positive in immunohistochemistry **(C)**.

The tumor was excised and the histopathological diagnosis was AMF. In the operation, a 4 cm incision was made on the left scrotum. Under the scrotal wall, a well encapsulated mass was found, approximately 5 cm × 4 cm in size. Because of the clear boundary of this tumor, the mass was easily separated from the adjacent tissues and the left testis remained intact after surgery. The left spermatic vessels were found to supply blood for the tumor and after ligating the tumor-feeding blood vessels, which were derived from spermatic vessels, the tumor was completely removed. At the end of the surgery, a drainage strip was left in the incision of the scrotum. Microscopically, the tumor was rich in thin-walled and small-size blood vessels (Figures 2A,B). Hyperplastic spindle cells could be seen around the vessels (Figure 2B). In immunohistochemical testing, the stains for muscle-specific actin (SMA) were positive and S100 was negative. Tumor cells displayed positive for desmin (Figure 2C), CD34, and Ki67 proliferation index of about 1%. Within 3 months of follow-up, this patient had no other discomfort and recovered well.

## Discussion

Angiomyofibroblastoma is a rare benign soft-tissue neoplasm that belongs to the mesenchymal tumor class (3, 4). Its clinical course is usually characterized by slow and painless growth (1). Complete surgical resection is the best treatment (3). Currently, the imaging characteristics of AMF are rarely reported and it is hard to be diagnosed just by MRI. The diagnosis of AMF always depends on pathology. Under the microscope, the plump spindled and epithelioid mesenchymal cells are inclined to gather around a large number of thin-walled small to medium-sized vessels and often contain mast cells (5, 6). Besides, mitoses in AMF tumor cells are low (5). In immunohistochemical analysis, desmin, progesterone receptors (PR), estrogen (ER), and vimentin are always positive, while S100 is negative and CD34 is rarely expressed (1, 5). It is reported that the expression rate of SMA in all cases is 12%, which could support the presence of myofibroblastic differentiation in AMF (5).

In our case, we found that the tumor was heterogeneous hyperintense on T2-weighted images. The mixed signal may be related to its content, including myxoid matrix, collagenous stroma, and spindle cells. On T1-weighted images, it was hypointense but we found some high-signal striped areas and blood vessel flow void signs. At first, we thought that the high-signal areas represented fat, but subsequent contrast-enhanced MRI confirmed that they were vessels. The tumor was markedly enhanced after injection of the intravascular contrast agent, and we found that the contrast-media could help to show the small vessels in the tumor. The enhancement pattern of the MRI findings is consistent with the other two female cases (7, 8), one in the posterior perivesical space and the other in the paravaginal space. These manifestations may correlate with the rich supply of capillary-like blood vessels in it, or due to the contrast medium is difficult to be quickly washed out from a large number of fibrous tissues (3, 7). Besides, the well-defined boundary and unrestricted diffusion on DWI might indicate a low-grade tumor.

Angiomyofibroblastoma should be differentiated from angiomyofibroblastoma-like tumor (AMF-like tumor, which can also be called cellular angiofibroma). AMF-like tumor was first defined by Laskin et al. (9) in 1998. The onset age in AMF-like tumors in men is usually higher than that in women. There are many similarities between AMF and AMF-like tumors in imaging manifestations, which may be due to some pathological similarities. On T2-weighted images, AMF-like tumors also show high non-homogeneous signals (10, 11). Whereas, on T1-weighted images, AMF-like tumors could present hyperintense foci because of the fat component. The reason may be that, pathologically, AMF-like tumor is more likely to contain adipocytes (in half of the cases), while AMF may have fewer mature adipocytes (10% of cases) (3). Thus, we speculate that, compared with AMF, an AMF-like tumor is more possible to present high-intensity areas on T1- weighted images. However, in another case, when the tumor is a lipomatous variant of AMF (there is prominent mature fat in the tumor), the high signal region might be easily visible on T1- weighted images (5).

Differential diagnosis also includes aggressive angiomyxoma. Aggressive angiomyxoma exhibits hyperintensity similar to that of AMF on T2-weighted images and showed non-homogeneous enhancement, but the difference is that a typical whorled signal intensity pattern could be seen on fat suppression T2-weighted and enhanced T1-weighted images, which is associated with the myxoid matrix with an internal swirl (12). Aggressive angiomyxoma does not infiltrate the surrounding structures but tends to grow around them (12).

In this case report, we propose some imaging signs that might be useful for the diagnosis of AMF. In fact, because of some non-specific features in MRI images, it is difficult to make an accurate diagnosis based on imaging alone. In this situation, pathology still plays an important role.

## Conclusion

Here, we display some imaging features of scrotal AMF, such as mixed high signal intensity on T2-weight images, obvious progressive enhancement, and small vessel on T1-weight images and enhanced images. We hope that the abovementioned findings can help identify AMF in the male genital tract. More literature on the imaging of AMF is needed to help confirm and summarize the imaging features of AMF.

## Data Availability Statement

The original contributions presented in the study are included in the article/supplementary material, further inquiries can be directed to the corresponding authors.

## Author Contributions

JZ and LZ: collected the information of the patient, data analysis and interpretation, and reviewed the literature. JZ: wrote the draft of the manuscript. LZ: contributed to the language revising and reviewed the manuscript. CS and LL: contributed to the conception and design of this case report. All the authors contributed to the article and approved the submitted version.

## Funding

This study was supported by the Guangzhou Key Laboratory of Molecular and Functional Imaging for Clinical Translation (Project No. 201905010003).

## Conflict of Interest

The authors declare that the research was conducted in the absence of any commercial or financial relationships that could be construed as a potential conflict of interest.

## Publisher's Note

All claims expressed in this article are solely those of the authors and do not necessarily represent those of their affiliated organizations, or those of the publisher, the editors and the reviewers. Any product that may be evaluated in this article, or claim that may be made by its manufacturer, is not guaranteed or endorsed by the publisher.
